# Modulation of Long-Term Potentiation by Gamma Frequency Transcranial Alternating Current Stimulation in Transgenic Mouse Models of Alzheimer’s Disease

**DOI:** 10.3390/brainsci11111532

**Published:** 2021-11-19

**Authors:** Won-Hyeong Jeong, Wang-In Kim, Jin-Won Lee, Hyeng-Kyu Park, Min-Keun Song, In-Sung Choi, Jae-Young Han

**Affiliations:** 1 Department of Physical & Rehabilitation Medicine, Chonnam National University Hospital, Gwangju City 61469, Korea; jwhiiii@hanmail.net (W.-H.J.); wangto9@naver.com (W.-I.K.); barksa37@naver.com (J.-W.L.); phk1118@naver.com (H.-K.P.); drchoiis@hanmail.net (I.-S.C.); 2 Department of Physical & Rehabilitation Medicine, Regional Cardiocerebrovascular Center, Center for Aging and Geriatrics, Chonnam National University Medical School & Hospital, Gwangju City 61469, Korea; drsongmk@daum.net

**Keywords:** transcranial alternating current stimulation, Alzheimer’s disease, noninvasive brain stimulation, gamma frequency, long-term potentiation, synaptic plasticity, transgenic mouse model, 5xFAD, excitatory postsynaptic potential, Western blot analysis

## Abstract

Transcranial alternating current stimulation (tACS) is a neuromodulation procedure that is currently studied for the purpose of improving cognitive function in various diseases. A few studies have shown positive effects of tACS in Alzheimer’s disease (AD). However, the mechanism underlying tACS has not been established. The purpose of this study was to investigate the mechanism of tACS in five familial AD mutation (5xFAD) mouse models. We prepared twenty 4-month-old mice and divided them into four groups: wild-type mice without stimulation (WT-NT group), wild-type mice with tACS (WT-T group), 5xFAD mice without stimulation (AD-NT group), and 5xFAD mice with tACS (AD-T group). The protocol implemented was as follows: gamma frequency 200 μA over the bilateral frontal lobe for 20 min over 2 weeks. The following tests were conducted: excitatory postsynaptic potential (EPSP) recording, Western blot analysis (cyclic AMP response element-binding (CREB) proteins, phosphorylated CREB proteins, brain-derived neurotrophic factor, and parvalbumin) to examine the synaptic plasticity. The EPSP was remarkably increased in the AD-T group compared with in the AD-NT group. In the Western blot analysis, the differences among the groups were not significant. Hence, tACS can affect the long-lasting enhancement of synaptic transmission in mice models of AD.

## 1. Introduction

Alzheimer’s disease (AD) is a neurological disease characterized by progressive cognitive decline resulting in memory deficit and behavioral changes [1,2,3]. It is prevalent in a majority of dementia cases. It has been estimated that about 110 million people worldwide will have the disease by 2050 [4]. Today, AD is a global burden, and this trend may continue unless an effective treatment is developed [5]. Currently, pharmacologic treatment is the main therapeutic modality for patients with AD; however, this therapeutic effect has been proven to be insufficient in ameliorating the state of patients affected by the disease. In addition, various side effects have been reported [6,7,8]. As an alternative to drug therapy, noninvasive brain stimulation (NIBS) has been studied in patients with AD. Previous studies have suggested that repetitive transcranial magnetic stimulation (rTMS) and transcranial direct current stimulation (tDCS) can be used for therapeutic purposes in patients with AD [9,10,11]. Furthermore, Hausner et al. demonstrated an improvement in the mini-mental state examination (MMSE) score after electroconvulsive treatment in patients with AD and major depressive disorder (MDD) [12]. Moreover, although there are no noninvasive vagus nerve stimulation (nVNS)-related human studies of AD to date, microglial modulation through nVNS was reported in an animal study (mouse model of AD) [13]. However, according to the studies published so far, cranial electrotherapy stimulation (CES), a type of NIBS, is known to be ineffective in improving cognitive function in the case of AD [14,15].

Recently, as a method of NIBS, transcranial alternating current stimulation (tACS) has been studied in patients with several diseases, including Parkinson’s disease and schizophrenia, to improve cognitive function [16,17]. In addition, previous studies showed that tACS can alleviate symptoms in other psychiatric diseases, such as anxiety disorder, MDD, and obsessive-compulsive disorder [18,19,20]. It is easy to apply in various conditions and a relatively inexpensive tool [21]. Since it is a noninvasive method, it has fewer side effects, including mild headache, nausea, and fatigue [22].

In a previous study using AD transgenic mouse models, neuronal activity, especially gamma frequency oscillations, was found to be impaired in the hippocampus [23]. This condition resulted from the amyloid-beta that alter the excitatory/inhibitory balance of the neuronal network and disrupt the inhibitory interneurons [24,25]. Moreover, tACS can manipulate neuronal oscillations, thereby influencing cognitive processes [26]. Ali et al. showed that tACS is more effective in the entrainment of brain waves using a specific frequency than tDCS, because human brain waves are more similar in form to alternating currents than direct currents [27].

Several studies have investigated the effectiveness of tACS in patients with AD [28,29,30,31]. Kehler and Moussavi et al. reported that, in patients with AD, tACS with brain exercise can be more beneficial for cognitive function than brain exercise alone [28,29]. Benussi et al. showed that tACS could enhance memory functions in patients with AD [30]. Dhaynaut et al. reported that tACS may modulate the Tau-related burden in AD measured by positron emission tomography (PET) [31]. Xing et al. suggested a protocol of tACS in patients with AD for a randomized controlled trial that has not yet been completed [32]. However, these studies were limited in terms of identifying the underlying mechanisms of action, because they involved human subjects. In the present study, we conducted a tACS experiment in transgenic mouse models of AD to elucidate its mechanism.

## 2. Materials and Methods

We first prepared the wild-type C57BL/6 female mice (Damul Science, Daejeon, Korea) and transgenic male mice expressing 5 familial AD mutations (5xFAD) carrying a Swedish double mutation (KM670/671NL) in the amyloid precursor protein (APP) gene. After crossbreeding them, we waited until the mice were 3 weeks old. At this time, only male mice were classified separately and underwent genotyping using polymerase chain reaction (PCR) with ear tissue. In this way, we confirmed the mice as 5xFAD or wild type. We then included twenty 4-month-old mice for the experiments.

### 2.1. Transcranial Alternating Current Stimulation

We prepared an oscillating-current stimulator (NT Brain 100, CyberMedic Corp., Iksan, Korea). Twenty mice were divided into four groups, with each group consisting of five mice: wild-type mice with no stimulation (WT-NT group), wild-type mice receiving tACS (WT-T group), 5xFAD mice with no stimulation (AD-NT group), and 5xFAD mice receiving tACS (AD-T group). The stimulation group received tACS with a gamma frequency (40 Hz) of 200 μA over the F3 and F4 (bilateral frontal lobe) areas for 20 min. During stimulation, isoflurane was used to minimize mice movements. tACS was then applied to the precise location. In this manner, the stimulus was implemented 10 times in 2 weeks for five consecutive days with two off days. The AD-NT and WT-NT groups did not undergo stimulation. Instead, they were caged without anesthesia for 20 min, which was equal to the stimulation time. 

### 2.2. Preparation of Brain Tissue

The brain was immediately extracted after the mice were euthanized under anesthesia after 2 weeks of starting the experiment. Artificial cerebrospinal fluid (aCSF) was prepared as follows to maintain osmolarity and pH at the physiological levels: 2.8-mM KCl, 125-mM NaCl, 1.25-mM NaH_2_PO_4_, 2-mM CaCl_2_, 1-mM MgSO_4_, and 26-mM NaHCO_3_. Furthermore, aCSF and freezing liquid were used to immerse the isolated brains. After placing the brains on a cooling pad, we found the exact location of the hippocampus using various brain landmarks such as the cerebellum and used a rotary slicer and automatic chopper (Mickle Laboratory Engineering Co. Ltd., Gomshall Guildford, UK). The thickness of each sample was about 400 μm. The slices were immersed in aCSF for at least an hour. During this process, oxygen was supplied to stabilized PH with carbogen (mixture of carbon dioxide (5%) and oxygen (95%)).

### 2.3. Excitatory Postsynaptic Potential (EPSP)

A glass-bottomed recording chamber (Glass cover slip CS-22/40, Warner Instruments, Holliston, MA, USA) filled with aCSF was prepared to record the EPSP in the sliced hippocampus. In this chamber, carbogen was also supplied. Using a nichrome recording electrode (Nickel/Chromium wire, Advent research materials Ltd., Oxford, UK), we recorded the field EPSP (fEPSP) in CA1 of the hippocampal subfield region. During this process, we delivered every pulse at 15-s intervals. For each waveform of fEPSP, the initial slope of the fEPSP was calculated, which showed the postsynaptic response. After adjusting the baseline fEPSP slope (30% of the maximal response), a stimulation was delivered at a high frequency (100 Hz) to the CA3–CA1 hippocampal synapses for long-term potentiation (LTP). The baseline fEPSP slope was recorded for 30 min, and LTP was recorded for 60 min. We analyzed the data using WinLTP software (WinLTP Ltd., Bristol, UK).

### 2.4. Western Blot Analysis

For protein extraction from the sliced hippocampus, the radioimmunoprecipitation assay buffer and protein inhibitor cocktail were used and centrifuged at 4 °C for 30 min at 15,000 rpm. After the spin down, the upper layer solution (protein) was obtained for protein quantification with the bicinchoninic acid assay. We prepared 10–12% gels and a polyvinylidene fluoride membrane for protein loading and transfer, respectively. The membrane was then incubated for blocking with 5% skim milk for 1 h. Then, using Tris-buffered saline with Tween 20 (TBST), we washed it (3 times). We prepared the diluted primary antibody solution (cyclic AMP response element-binding (CREB) proteins (1:1000), phosphorylated CREB (pCREB) proteins (1:1000), brain-derived neurotrophic factor (BDNF) (1:1000), and parvalbumin (PV) (1:1000)) and incubated them overnight with the membranes (4 °C). The next day, using TBST, we washed the membranes (3 times). We also incubated the diluted secondary antibody solution (rabbit immunoglobulin G (IgG; 1:2000)) with the membrane for one and a half hours. In the same way, we washed the membranes using TBST (3 times). The membranes were incubated in a chemiluminescent substrate for horseradish peroxidase for detection. The Western blot was analyzed using UVITEC Mini HD9 (Alliance UVItec Ltd., Cambridge, UK). 

### 2.5. Statistical Analyses

We used the Kruskal–Wallis test to compare the differences between the AD-NT, AD-T, WT-NT, and WT-T groups. A post hoc analysis was performed by the Bonferroni method. The significance level for multiple comparisons after the Bonferroni method was 0.0083. There was a system that provided a new report of the adjusted *p*-value based on the significance level of 0.05 after Bonferroni correction in the SPSS program, version 27.0 (IBM, SPSS, Armonk, NY, USA). Therefore, we set a statistical significance level of 0.05 based on the adjusted *p*-value. A data analysis was conducted through SPSS, version 27.0 (IBM, SPSS, Armonk, NY, USA).

## 3. Results

### 3.1. fEPSP Responses

**Figure 1:**
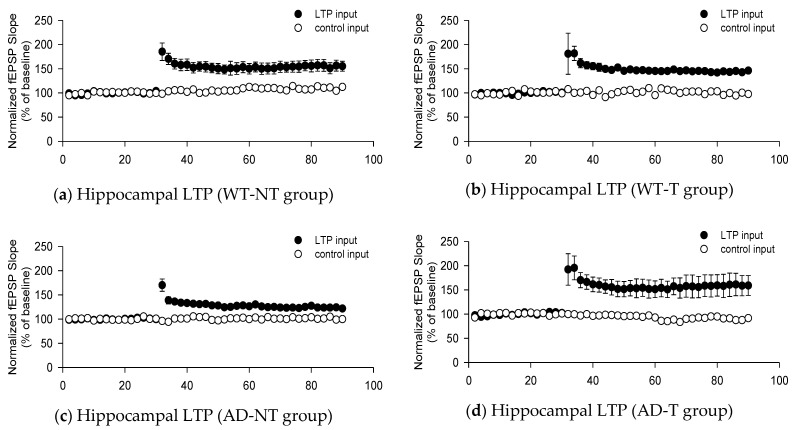
The graphs show the field excitatory postsynaptic potential (fEPSP) responses in each group. High-frequency stimulation are delivered at 30 min for long-term potentiation (LTP). (**a**) fEPSP in the hippocampal region of wild-type mice that did not receive transcranial alternating current stimulation (tACS). (**b**) fEPSP in the hippocampal region of wild-type mice that received tACS. (**c**) fEPSP in the hippocampal region of 5 familial AD mutation (5xFAD) mice that did not receive tACS. (**d**) fEPSP in the hippocampal region of 5xFAD mice that received tACS. The white dots are the baseline fEPSP slopes, and the black dots are the main results of the fEPSP slope stimulated by high frequency. The fEPSP slope was remarkably increased in the AD-T group (**d**) compared to that in the AD-NT group (**c**) (*p*-value = 0.003). In addition, the fEPSP slope was higher in the WT-T group (**b**) (*p*-value = 0.001) and WT-NT group (**a**) (*p*-value = 0.000) than in the AD-NT group (**c**). However, there were no significant differences among the other groups. The data from each group show mean values and standard errors (SE). For statistical analysis, the Kruskal–Wallis test and the Bonferroni method for post hoc analysis were used.

The fEPSP slope showed the responses of the WT-NT group (159 ± 10%), WT-T group (145 ± 4%), the AD-NT group (123 ± 3%), and the AD-T group (156 ± 20%). The fEPSP slope was remarkably increased in the AD-T group compared to that in the AD-NT group (*p*-value = 0.003). In addition, the fEPSP slope was higher in the WT-T group (*p*-value = 0.001) and WT-NT group (*p*-value = 0.000) than in the AD-NT group. However, there were no significant differences among the other groups (Figure 1).

### 3.2. Protein Level Analyzed by Western Blot Analysis

**Figure 2:**
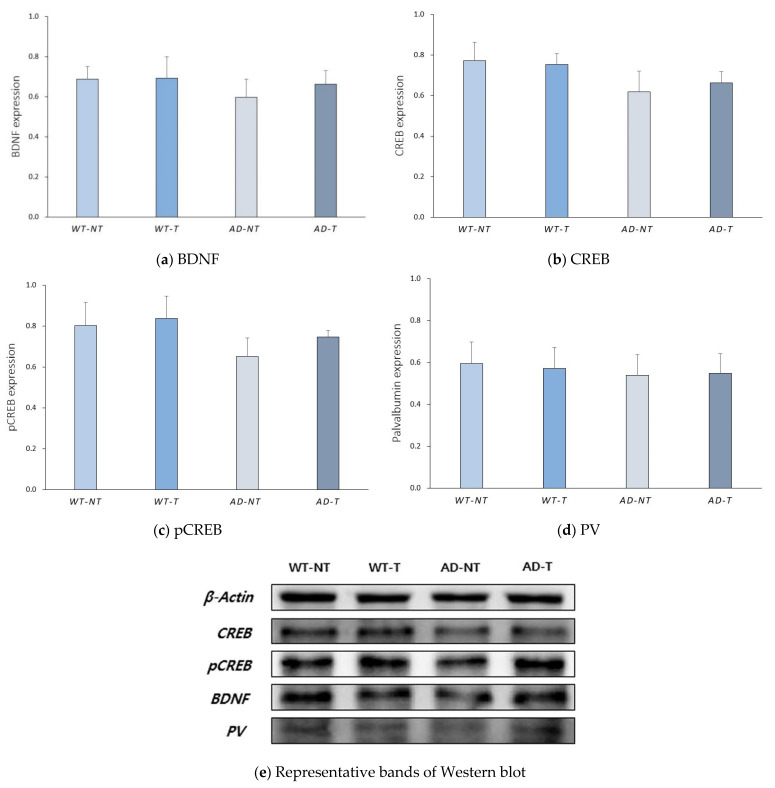
Western blot analysis of brain-derived neurotrophic factor (BDNF), cyclic AMP response element-binding protein (CREB), phosphorylated CREB (pCREB), and parvalbumin (PV). (**a**) Quantitative BDNF expression level in the WT-NT group (0.69 ± 0.06), the WT-T group (0.69 ± 0.11), the AD-NT group (0.60 ±0.09), and the AD-T group (0.66 ± 0.07). (**b**) Quantitative CREB expression level of the WT-NT group (0.77 ± 0.09), the WT-T group (0.75 ± 0.05), the AD-NT group (0.62 ± 0.10), and the AD-T group (0.66 ± 0.06). (**c**) Quantitative pCREB expression level of the WT-NT group (0.80 ± 0.11), the WT-T group (0.84 ± 0.11), the AD-NT group (0.65 ± 0.09), and the AD-T group (0.75 ± 0.03). (**d**) Quantitative PV expression level of the WT-NT group (0.60 ± 0.10), the WT-T group (0.57 ± 0.10), the AD-NT group (0.54 ± 0.10), and the AD-T group (0.55 ± 0.09). (**e**) Representative bands of CREB, pCREB, BDNF, and PV. The data from each group show the mean values and standard errors (SE). A statistical analysis was performed using the Kruskal–Wallis test and the Bonferroni method for post hoc analysis.

A Western blot analysis was conducted to examine whether there were any changes in the amount of proteins related to neuroplasticity and gamma oscillation. There were no significant differences in the levels of BDNF, CREB, pCREB, and PV among the groups (Figure 2).

## 4. Discussion

Gamma frequency oscillations are prominent in the hippocampus, which is a major region for the formation of memory [33]. In this region, gamma oscillation can be functionally classified into slow gamma oscillation (25–55 Hz) and fast gamma oscillation (60–100 Hz). Specifically, fast gamma oscillation is related to memory encoding, while slow gamma oscillation is related to memory retrieval [34]. Previous studies have suggested that patients with AD show reduced slow gamma oscillation and the ability to retrieve memory rather than a decreased ability to encode memory [23,35]. Iaccarino et al. showed a reduced hippocampal gamma waveform in transgenic 5xFAD mice and presented an experiment in which amyloid-beta production decreased after using visual stimulation (light flicker) with gamma frequencies [36]. Anthony et al. showed improved memory function and reduced hippocampal amyloid-beta in 5xFAD mice after using auditory tone stimulation with gamma frequencies [37]. In addition, several human studies have suggested that gamma tACS can be a new treatment strategy in patients with AD [28,29,30].

In this animal study, we investigated the mechanism of tACS in a transgenic mouse model. Oakley et al. reported that, in 5xFAD mice, cognitive function, including working memory, decreased in 4 months due to neurodegeneration [38]. Therefore, we waited until the mice were 4 months old before conducting the tests. We then implemented fEPSP to explore the mechanisms for changes occurring after applying the tACS. Previous studies have shown that high-frequency stimulations of mice’s hippocampal CA1 can be used for estimating the degree of synaptic plasticity, such as LTP [39]. LTP is a process in which there is increased synaptic strength, which is the signal transmission between a presynaptic neuron and a postsynaptic neuron. In response to the stimulus, α-amino-3-hydroxy-5-methyl-4-isoxazolepropionate receptor (AMPAR) trafficking plays the most decisive part in LTP through mobilizing preexisting AMPARs to the synaptic sites, resulting in increased AMPAR-mediated synaptic responses [40]. BDNF can upregulate the level of AMPAR and increase AMPAR trafficking in the hippocampus [41]. In addition, BDNF can induce LTP by increasing the synaptic response to high-frequency stimulation [42]. Besides the functional modification, BDNF modulates the structure by increasing the dendritic spine and arborization, which enhances the synaptic transmission [43]. In patients with AD, AMPAR is downregulated in an amyloid-beta-dependent manner, and BDNF is downregulated in the hippocampus and cortex, according to a recent meta-analysis [44,45]. Moreover, CREB can modulate the expression of BDNF promoters as a transcriptional factor. However, patients with AD show a decreased level of pCREB (activated form), reducing CREB activity, which is eventually caused by the decreased BDNF downregulation [46]. Hence, we measured the functional changes from an electrophysiological perspective and the amount of protein changes from a molecular perspective to determine the post-treatment effect of tACS. The electrophysiological test we conducted showed that the fEPSP slope was remarkably higher in the AD-T group than in the AD-NT group. However, there were no statistically significant differences between the WT-T and the WT-NT groups, suggesting that applying tACS in AD transgenic mice can increase the degree of synaptic plasticity. In the Western blot analysis, there were no statistically significant differences in the levels of BDNF, CREB, and pCREB among the groups.

In addition to investigating the CREB, pCREB, and BDNF levels, we also investigated the PV levels using the Western blot analysis. PV is a protein that affects the amplitude of the action potential. In addition, a positive PV interneuron is suggested to play an important role in the generation of gamma oscillation [47]. Moreover, Olivier et al. showed that PV can modulate short-term neuroplasticity, which was maintained for up to a few minutes, unlike long-term neuroplasticity [48]. In various mouse models of AD, including the 5xFAD, the density of PV-positive neurons decreased in the hippocampus [49,50]. Hence, we conducted a Western blot analysis of PV to identify whether external gamma oscillation application affected the change in the level of PV; there were no statistically significant differences in the PV levels among the groups.

The application of tACS in the transgenic mouse model could enhance the neuroplasticity at the electrophysiologic level, while the differences were not significant at the protein level. In other words, the synaptic strength was strengthened, but the upregulation of proteins related to neuroplasticity was not very significant.

## 5. Limitations and Future Directions

The limitation of this study was the lack of behavior tests among the groups, although genotyping identified each mouse type. In addition, compared to the positive results of EPSP, there were no differences among the groups in the Western blot analysis. This might have been the case because the treatment period was as short as 10 times, and this was not long enough to allow the proteins to be sufficiently upregulated. Next, although a nonparametric test was used for the statistical analysis, the small sample size may also have affected the results. Finally, unlike humans, animals move constantly during experiments, so we had no choice but to anesthetize them, because they had to be fixed in place for electrical stimulation. However, it has been reported that exposure to isoflurane for 2 h may be associated with decreased BDNF expression [51]. Furthermore, Sen et al. reported that exposure to isoflurane for 2 h may affect the inactivation of CREB and deteriorate cognitive function [52]. Although the anesthesia time in our experiment was as short as 30 min, its impact should also be considered, because anesthesia was not performed in the WT-NT and AD-NT groups. Therefore, in future studies, animal experiments using anesthetics other than isoflurane will be required when conducting tACS experiments related to AD. Further research will also be needed to explore the most appropriate tACS protocols, including stimulation time, by increasing the sample size.

## 6. Conclusions

In this study, we confirmed the enhancement of synaptic plasticity in transgenic mice of AD via an electrophysiologic test through the stimulation of gamma tACS in the frontal lobe. These findings support the results in previous human studies that tACS can improve cognitive function in patients with AD. Importantly, we presented the basis for applying tACS, a new treatment method that improves cognitive function in patients with AD. However, since there were no significant differences among the groups in the expressions of the proteins we observed during the Western blot analysis, it is necessary to investigate other pathways related to synaptic plasticity in the future. To the best of our knowledge, since there have been no studies with animal experiments using tACS in AD, further animal studies are needed to identify other unknown mechanisms in the effectiveness of tACS.

## Data Availability

The data presented in this study are available from the corresponding author upon reasonable request.
